# *Bacillus subtilis* spores as delivery system for nasal *Plasmodium falciparum* circumsporozoite surface protein immunization in a murine model

**DOI:** 10.1038/s41598-022-05344-2

**Published:** 2022-01-27

**Authors:** Maria Edilene M. de Almeida, Késsia Caroline Souza Alves, Maria Gabriella Santos de Vasconcelos, Thiago Serrão Pinto, Juliane Corrêa Glória, Yury Oliveira Chaves, Walter Luiz Lima Neves, Andrea Monteiro Tarragô, Júlio Nino de Souza Neto, Spartaco Astolfi-Filho, Gemilson Soares Pontes, Antônio Alcirley da Silva Balieiro, Rachele Isticato, Ezio Ricca, Luis André M. Mariúba

**Affiliations:** 1 Programa de Pós-Graduação Stricto Sensu em Biologia Celular e Molecular do Instituto Oswaldo Cruz (IOC/Fiocruz), Rio de Janeiro, RJ Brazil; 2 Instituto Leônidas e Maria Deane, Fiocruz Amazônia, Manaus, Brazil; 3 Programa de Pós-Graduação em Biotecnologia, Instituto de Ciências Biológicas, Universidade Federal do Amazonas (UFAM), Manaus, AM Brazil; 4 Programa de Pós-Graduação em Imunologia Básica e Aplicada, Instituto de Ciências Biológicas, Universidade Federal Do Amazonas (UFAM), Manaus, AM Brazil; 5 Fundação Hospitalar de Hematologia e Hemoterapia do Amazonas, HEMOAM, Manaus, AM Brazil; 6 Instituto Nacional de Pesquisa da Amazônia – INPA, Manuas, AM Brazil; 7 Department of Biology, Federico II University, Naples, Italy; 8 Centro Universitário Fametro, Manaus, AM Brazil; 9 Programa de Pós-Graduação Stricto Sensu em Biologia Parasitária do Instituto Oswaldo Cruz (IOC/Fiocruz), Rio de Janeiro, RJ Brazil; 10 Programa de Pós-Graduação Stricto Sensu em Ciências Aplicadas à Hematologia PPGH, Universidade do Estado do Amazonas (UEA), Manaus, AM Brazil; 11 Centro de Apoio Multidisciplinar (CAM), Universidade Federal do Amazonas (UFAM), Manaus, AM Brazil; 12 Instituto de Ciências Biológicas (ICB), Universidade Federal do Amazonas (UFAM), Manaus, AM Brazil

**Keywords:** Immunology, Microbiology, Molecular biology

## Abstract

Malaria remains a widespread public health problem in tropical and subtropical regions around the world, and there is still no vaccine available for full protection. In recent years, it has been observed that spores of *Bacillus subtillis* can act as a vaccine carrier and adjuvant, promoting an elevated humoral response after co-administration with antigens either coupled or integrated to their surface. In our study, *B. subtillis* spores from the KO7 strain were used to couple the recombinant CSP protein of *P. falciparum* (r*Pf*CSP), and the nasal humoral-induced immune response in Balb/C mice was evaluated. Our results demonstrate that the spores coupled to r*Pf*CSP increase the immunogenicity of the antigen, which induces high levels of serum IgG, and with balanced Th1/Th2 immune response, being detected antibodies in serum samples for 250 days. Therefore, the use of *B. subtilis* spores appears to be promising for use as an adjuvant in a vaccine formulation.

## Introduction

Malaria remains a serious public health problem worldwide and causes high morbidity and mortality in tropical and subtropical regions. In 2019, 229 million cases of malaria and 409 thousand deaths were registered worldwide^1^, with *Plasmodium falciparum* being responsible for the majority of these deaths^2^.

As yet, there is no effective vaccine to combat malaria, though there is a promising candidate, namely the RTS’S vaccine, which targets the pre-erythrocytic stage of the parasite^3^. The RTS’S vaccine comprises part of the central repeat domain and the C-terminal region of the *P. falciparum* circumsporozoite surface protein (*Pf*CSP) and presents T-cell epitopes fused to hepatitis B surface antigen^4^. RTS’S is the first malaria vaccine to reach phase III of clinical trials. The tests were carried out among children aged 5–17 months, who received three doses of the vaccine. The children showed a reduction of 39% in mild and 31.5% in severe cases of malaria. However, this partial protection tends to decrease with time, and its effectiveness is age-dependent^5^.

Recently, a new vaccine candidate for malaria, known as R21, has shown promising results in clinical trials. R21 has the same CSP sequence as the RTS'S vaccine, though a different adjuvant formulation. Preliminary results from the phase II clinical trial carried out with children aged 5–17 months demonstrated that the R21 vaccine has a long-term efficacy of 77%^6^. The aforementioned study demonstrated the important role of adjuvants in vaccine efficacy. Therefore, the search for new adjuvants is crucial to the development of effective vaccines since the increase of antigen immunogenicity causes the immune response to provide long-term protection^7^.

*Bacillus subtilis* spores have proven to be a valuable tool for stimulating stronger immune responses by enhancing antigen presentation and T cell priming^8–10^. After the integration of antigens in the surface of spores by coupling or recombination, it was observed that they act as adjuvants in different routes of administration, and stimulate the production of pro-inflammatory cytokines and the recruitment/maturation of dendritic cells^11–13^. In addition, these spores can induce high levels of IgA and IgG neutralizing antibodies and amplify the cellular response of T CD4^+^/CD8^+^ antigen-specific cells^14,15^. Other studies have demonstrated that *B. subtilis* is also recognized by TLR2, TLR4, and TLR9 and can induce Th1/Th2 responses, with the presence of IgG2a and IgG1 in immunized mice sera^16–18^.

Therefore, since *Bacillus subtilis* spores can act as a remarkable carrier for antigen delivery, we present here the first description of the use of *B. subtilis* spores as a novel adjuvant strategy for intranasal vaccination against malaria in a murine model. The recombinant *Pf*CSP (r*Pf*CSP) production and the analysis of the humoral response (total IgG and subclasses) of the immunized animals against this antigen are described.

## Materials and methods

### Recombinant *Plasmodium falciparum* CSP production

For the design of the recombinant protein of *P. falciparum* CSP (r*Pf*CSP), we used the sequence proposed by Stoute et al*.* (1997)^19^ present in commercial malaria vaccine RTS’S. The synthetic gene was produced by the Thermofisher company. It presents an improvement of the codons for expression in *E. coli* and was inserted in pRSET “A” expression vector. The expression and purification of the recombinant protein were carried out following the methodology described by Souza et al*.* (2014)^20^. The *E. coli* BL21 (DE3) *pLysS* strain was transformed with this construction and induced to produce the recombinant protein using IPTG (isopropyl β-D-1-thiogalactopyranoside), at a final concentration of 1 mM, for 3 h at 37 °C in Luria Bertani medium, containing the antibiotics chloramphenicol (11.4 µg/mL) and ampicillin (100 µg/mL). Protein purification was performed using the NTA nickel column (QIAGEN), following the guidelines described by the manufacturer. In order to analyze the expression and purification of the r*Pf*CSP protein, SDS-PAGE was performed following the protocol of Maniatis et al*.* (1989)^21^. Protein mass in SDS-PAGE was determined using the iBright analysis software (Thermo Fisher Connect™). After purification, immunoblots were performed using r*Pf*CSP on a nitrocellulose membrane, and a monoclonal antibody (mAb) for detection of the 6xHis tag (recognized sequence HHHHHHG, Sigma-Aldrich, cat. No. MA1-21315) and an mAb against *P. falciparum* CSP (recognized sequence NANPNVDPNANP, kindly provided by BEI Resources, cat. No. MRA-183A 2A10), as the primary antibody. Developing was performed using an anti-IgG mouse coupled with horseradish peroxidase (KPL, cat. No. 215-1802) and 3,3'-diaminobenzidine (DAB, Sigma-Aldrich. cat. No. D7304).

### Preparation and quantification of *B. subtilis* spores

The spores of *Bacillus subtilis*, strain KO7, were obtained by the nutrient exhaustion method, using Difco sporulation medium, at 37 ºC, under constant agitation for 72 h. Afterwards, the spores were centrifuged at 4,000 rpm for 20 min, washed twice with Milli-Q water, and left for 16 h at 4 °C. The sample was deactivated by autoclaving at 121 °C, for 45 min. Spore quantification was performed by flow cytometry using FACSCanto (BD) and a Trucount kit (BD), following the manufacturer’s guidelines and the method described in Patent US20040023319A1 (supplementary figure 1).

### Coupling of r*Pf*CSP to the spores’ surface

Coupling was performed according to the method described by Falahati-pour et al*.*^22^. The *B. subtilis* spores at 1 × 10^8^ were resuspended with 250 µL of 1-ethyl-3-(3-dimethylaminopropyl) carbodiimide (EDC) (5 µg/ml) and left at room temperature for 15 min. Then, 250 µL of N-hydroxysuccinimide (NHS) (5 µg/ml) were added, and the sample was incubated at 4 ºC for 30 min under agitation. Afterwards, the samples were centrifuged and placed in contact with 10 µg of r*Pf*CSP for 16 h at room temperature, under constant agitation. Subsequently, the spores were then washed 3 times with 0.01 M phosphate saline buffer (PBS), and finally resuspended in 600 µL of PBS, and were stored at 4 °C until the moment of use. The dot blot method was used to quantify the remaining protein in the supernatant after the coupling assay. A curve with varying amounts of supernatant of the r*Pf*CSP + S*Bs*KO7 coupled sample (25 µl, 12 µl, 6 µl, 3 µl), as well as dilutions of purified r*Pf*CSP protein (2 µg; 1 µg; 0.5 µg; 0.25 µl; 0.125 µl; 0.06 µl) to be used as the standard, were applied under vacuum to the nitrocellulose membrane (Amersham™ Protran®) using the Bio-Dot device (BIO-RAD). The nitrocellulose membrane was blocked in 5% bovine serum albumin BSA solution dissolved in PBS 1 × for 1 h at room temperature. The membrane was subjected to washings with PBS 1x-Tween 80 at 0.05% and incubations with anti-CSP monoclonal antibody (BEI Resources, cat. No. MRA-183A 2A10) at a 1:1000 ratio and anti-mouse IgG secondary antibody conjugated to phosphatase alkaline (Phosphatase-KPL) in the proportion 1:10,000 for 1 h. Detection was performed with the chromogen of the *WesternBreeze*® kit (Thermo-Fisher Scientific) following the manufacturer’s recommendations. Sample quantification was obtained after scanning and analyzing the membrane image using the program iBright analysis software (Thermo Fisher Connect™). Based on a protein concentration curve, it was determined the total amount of remaining r*Pf*CSP in the specific volume of supernatant. Subtracting the original amount of protein used (10 µg) and the remaining amount, it was determined the average percentage of protein which coupled to *Bacillus subtilis* spore surface.

### Nasal immunization

The nasal immunization regime and experimental design was based on Santos et al*.* (2020)^13^, with some alterations made by our group. A total of 25 female animals (*Mus musculus Balb/c*) were used and divided into 5 groups containing 5 animals in each: (1) 10 µg r*Pf*CSP coupled to *B. subtilis* spores at 1 × 10^8^ (r*Pf*CSP + SBsKO7); (2) only 10 µg r*Pf*CSP; (3) Immunization only with spores of *B. subtilis* 1 × 10^8^(SBsKO7); (4) Immunization only with 0.01 M PBS; and (5) Unimmunized mice. Mice were intranasally vaccinated on days 0, 14 and 21 of the experiment**.** The study was authorized by the Ethics Committee on Animal Use of the National Institute for Amazonian Research (CEUA-INPA) under number 031/2018 according to international recommendations for ethics in animal experimentation (ARRIVE guidelines) and by guidelines for animal use and care based on the standards established by National Council for the Control of Animal Experimentation (CONCEA). Enzyme-linked immunosorbent assays (ELISA) were used for the evaluation of the humoral response. For this, blood samples were collected on days zero (D0), D14, D21 and D35 in all groups, and D50, D100, D150, D200 and D250 in groups that presented antibody titers detected by ELISA until D35 (Fig. 2A).

### Indirect ELISA

96-well plates were sensitized with r*Pf*CSP, which was diluted in carbonate buffer (pH 9.6) and incubated overnight at 4 ºC. Next, plates were washed with 0.01 M PBS/0.05% of Tween 20, and the wells were blocked using 10 mM PBS/2.5% BSA at 37 ºC, for 1 h. The mice sera were diluted at 1:100 in 10 mM PBS/2.5% BSA, which was added to the wells, and incubated at 37 ºC for 1 h. Then, the plates were washed 4 times with 10 mM PBS/0.05% Tween 20, and the secondary antibody (anti-IgG mouse HRP conjugate, ZyMax) was added at 1:2000 dilution, and subsequently incubated at 37 ºC, for 1 h. The plates were washed again, and the development was carried out with a chromogenic substrate (Scienco One Step—TMB), which was added to each well for 20 min. Finally, the reaction was interrupted with H_2_SO_4_ (2 M). The optical density (OD) was determined using an ELISA plate reader (Molecular Devices-FlexStation 3) with a 450 nm filter. The same procedure was performed for the detection of IgG antibody subclasses, and the secondary antibody was replaced with isotype-specific antibodies against mouse IgG1, IgG2a, IgG2b and IgG3 (Sigma-Aldrich®), which was diluted according to the manufacturer’s instructions.

### Statistical analysis

The results were analyzed using the mixed linear models in order to determine the variations between the groups of animals. All analyses were performed using R software version 4.0.2, and R studio version 1.1.4. The significance level considered was p < 0.05. Cutoff was calculated with the negative controls mean plus two and three times of its standard deviation. Box plot graphics were prepared to represent negative controls reactivity.

## Results

### Recombinant *Plasmodium falciparum* CSP was successfully expressed and purified from the soluble phase

**Figure 1:**
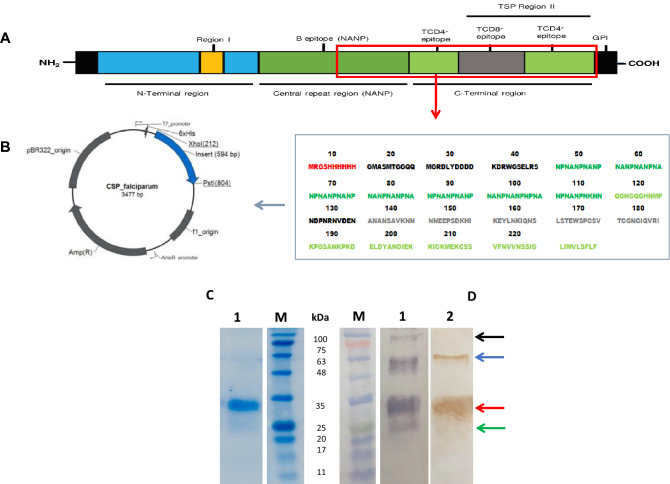
Construction and confirmation of the expression of the rPfCSP. (**A**) A graphical representation of CSP. The region corresponding to the construction of the r*Pf*CSP developed in this study is highlighted; (**B**) Amino acid sequence corresponding to r*Pf*CSP and the expression plasmid in which it was inserted (pRSET A, Invitrogen); (**C**) SDS-PAGE analysis of recombinant protein, BLUeye Pre-stained ladder (Sigma-Aldrich) (lane M), and electrophoretic profile of r*Pf*CSP elution (lane 1); (**D**) western blot confirming the r*Pf*CSP antibodies recognition. Lane 1: anti-HisG monoclonal antibody recognition. Black, blue, red, and green arrows indicate ~ 120, 63, 33, and 25 kDa proteins, respectively. Development step was performed using BCIP/NBT chromogenic substrate from Western breeze kit (Invitrogen); Lane 2: anti-*Pf*CSP monoclonal antibody (BEI Resources, cat. No. MRA-183A 2A10) recognition. Blue, red, and green arrows indicate 63, 33, and 25 kDa proteins, respectively. Development step was performed using 3,3'-diaminobenzidine (DAB, Sigma-Aldrich. cat. No. D7304).

The recombinant CSP from *Plasmodium falciparum* (r*Pf*CSP) used in this study comprises part of the central or repeat region (NANP), and the entire C-terminal region without the GPI anchor (Fig. 1A). Electrocompetent *Escherichia coli*, strain BL21 (DE3) p*LysS*, were transformed with the expression plasmid pRSET A containing the r*Pf*CSP gene sequence (Fig. 1B). The designed protein was successfully expressed and purified along with a polyhistidine tag and was present in the soluble portion of the bacterial lysate. Around 0.5 to 1 mg per liter of bacteria culture was obtained. After induction, the protein had an apparent molecular mass of around 33 kDa (Fig. 1C, Supplementary figure 2A). Other higher-mass proteins around 120 kDa and 63 kDa were also observed (lane 2, Fig. 1D). Immunoblot using anti-6xHIS tag (line 1, Fig. 1D,) and anti-*Pf*CSP monoclonal antibodies (Lane 2, Fig. 1D, Supplementary figure 2B,C) recognized r*Pf*CSP in the apparent molecular mass and in higher-mass proteins, confirming that all of them correspond to the designed recombinant *Pf*CSP.

### Adsorption of recombinant *Pf*CSP on *B. subtilis* spores

1 × 10^8^ purified spores of *B. subtilis* were used to adsorb 10 µg of r*Pf*CSP following Santos et al. (2020)^13^ with 1.0 × 10^6^ spores/gram. The efficiency of adsorption was evaluated by dot blotting as previously reported^8^^.^ The analysis revealed that about 50–65% of the r*Pf*CSP used in the reaction were stably adsorbed to spores (Supplementary figure 3). Based on the results of the dot blot analysis, 1 × 10^8^ adsorbed spores were used to nasally inoculare mice, therefore delivering 5–6.5 µg of r*Pf*CSP per dose to each of the immunized animal.

### *Bacillus subtilis* spores coupled to r*Pf*CSP were capable of inducing IgG production via intranasal immunization

**Figure 2:**
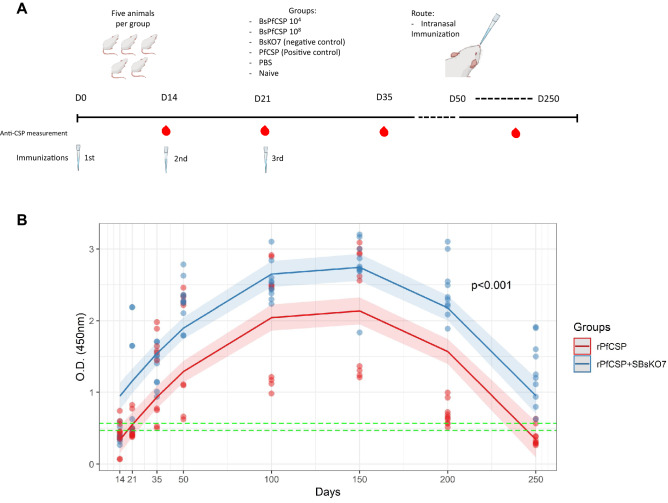
Indirect ELISA quantification of total IgG in mice. (**A**) Schematic showing the nasal immunization regimen and the follow-up period of the humoral immune response in mice. (**B**) Graph showing indirect ELISA quantification of total IgG from mice immunized intranasally with r*Pf*CSP and r*Pf*CSP coupled to S*Bs*KO7 (O.D 450 nm) at each blood sample collection day. The horizontal green lines correspond to the cutoff, which was calculated with the negative controls mean (non-immunized, 1 × 10^8^ S*Bs*KO7, and PBS mice groups) plus two/three times of its standard deviation. The blue line corresponds to the mean O.D. (450 nm) of the group of mice immunized with r*Pf*CSP + S*Bs*KO7 for each collection day (D14, D21, D35, D50, D100, D150, D200, and D250) and each blue dot represents the mean of O.D. of a mouse from the group in duplicate. While the red line corresponds to the mean O.D of the group of mice immunized only with r*Pf*CSP and each red dot corresponds to the mean O.D. of ELISA reactivity of a mouse blood sample. It is observed that the confidence bands do not touch each other at any time in the curves for each group, thus demonstrating that the r*Pf*CSP + S*Bs*KO7 group has the highest mean O.D. on all days of the experiment with a significant value of *p* < 0.001. The significance level considered was *p* < 0.05.

The r*Pf*CSP + SBsKO7 group induced high levels of serum IgG anti-r*Pf*CSP on D14 and reached the highest levels of IgG on D100 and D150.The group immunized with only r*Pf*CSP also induced the production of IgG. However, a detectable level of IgG anti-r*Pf*CSP was only observed on D35 and reached the highest levels also on D100 and D150, though these were significantly lower when compared to the r*Pf*CSP + SBsKO7 group (Fig. 2B). No significant concentration of IgG anti-r*Pf*CSP was detected in the negative controls (SBsKO7, PBS, and unimmunized group) (Supplementary figure 5A).

Anti-r*Pf*CSP titers were significantly higher in the r*Pf*CSP + SBsKO7 group on D14, D21, D35, D50, D200 and D250, when compared to the r*Pf*CSP group (Fig. 2B). Predictions made via statistical analysis demonstrated that the r*Pf*CSP + SBsKO7 and r*Pf*CSP groups presented anti-r*PF*CSP at the same level of negative control on D262 and D243, respectively. These results indicate that the r*Pf*CSP delivered through *B. subtilis* spores induces the production of longer-lasting antibodies.

### IgG subclass profiles

**Figure 3:**
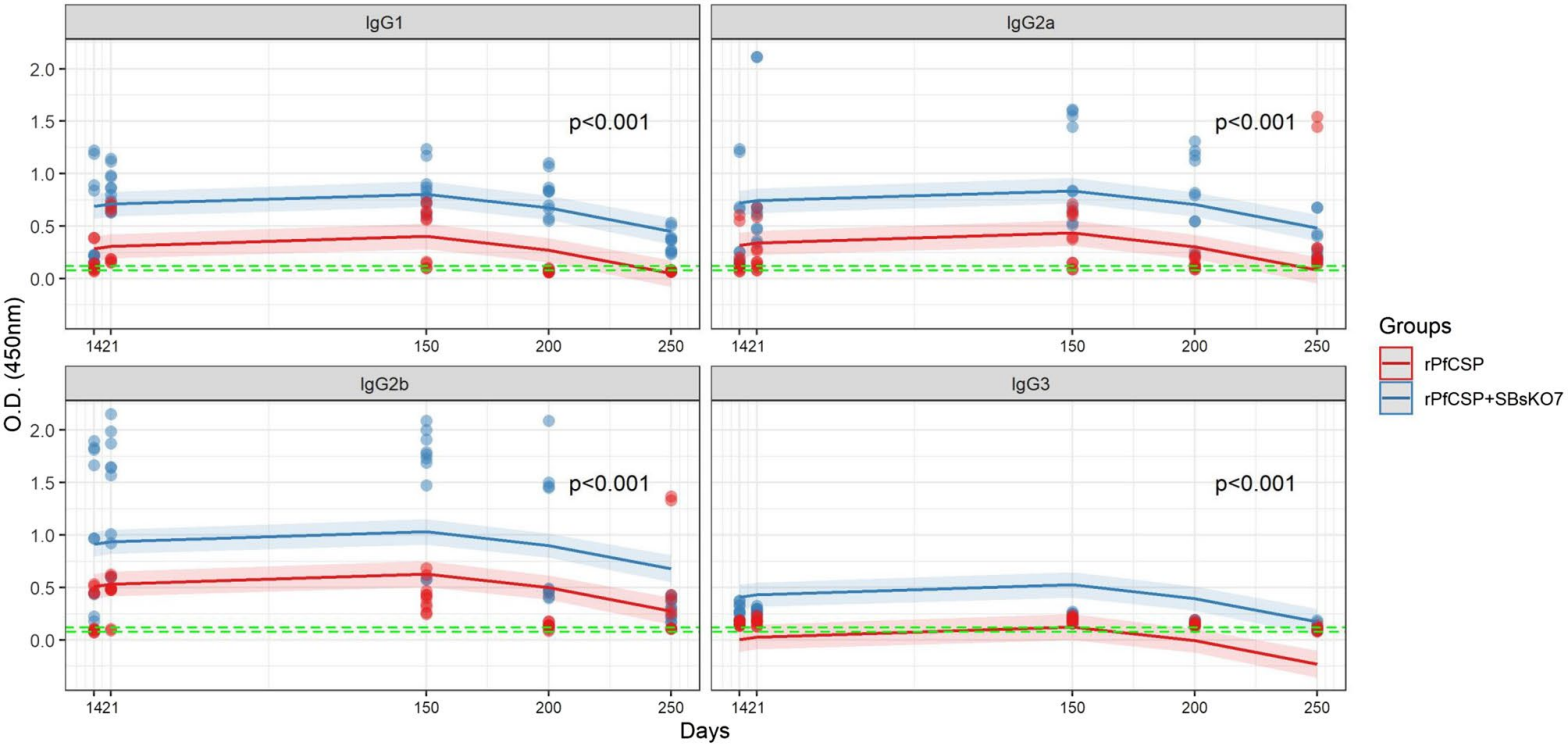
Indirect ELISA quantification of IgG subclasses in groups immunized only with r*Pf*CSP and with r*Pf*CSP + S*Bs*KO7. The green horizontal lines correspond to the negative control variation (S*Bs*KO7 1 × 108 and PBS). Each graph represents the quantification of the IgG1, IgG2a, IgG2b, and IgG3 subclasses of the r*Pf*CSP (red line) r*Pf*CSP + S*Bs*KO7 (blue line) mice groups. Each point corresponds to the mean O.D. (450 nm) of each mouse in the groups for the subclasses, in the previously mentioned colors. The significance level considered was *p* < 0.05. Only IgG2a was not significant when compared to IgG1 and, among the other subclasses, the *p* value was significant over time.

The r*Pf*CSP + SBsKO7 group showed the highest serum levels for IgG1, IgG2a, IgG2b and, IgG3 subclasses on D14, D21, D150, D200, and D250 when compared to the r*Pf*CSP group (Fig. 3). The subclass IgG2b showed the highest levels in both groups, followed by IgG2a, IgG1, and IgG3. The difference between the r*Pf*CSP + SBsKO7 and r*Pf*CSP groups observed in the IgG subclasses were statistically significant over the 250 days, except in the case of IgG1 and IgG2a (IgG2b > IgG2a = IgG1 > IgG3) (Supplementary figure 5B).

## Discussion

The *Pf*CSP was the first malaria protein cloned and recommended for use as an immunogen in malaria vaccine candidates^23^. Since then, significant efforts have been made to investigate the functional characteristics of this protein^24^. In this study, we produced a recombinant form of the PfCSP, which presented bands near and above the predicted molecular weight in SDS-PAGE analysis. All bands were recognized by anti-*Pf*CSP monoclonal antibodies, including the bands higher than expected, which, probably correspond to some r*Pf*CSP that may not denature completely. Noe et al*.* (2014)^25^ also reported the presence of a band at approximately 120 kDa after purification of a recombinant CSP, which was reduced when the SDS-PAGE denaturation buffer was changed. Thus, the authors concluded that the band corresponded to a dimeric form of recombinant CSP. Other studies have also reported the presence of bands above the initially predicted mass during the expression of r*Pf*CSP, indicating that there is significant interaction between r*Pf*CSP units even in a reduced condition^26,27^.

Our findings showed that *Bacillus subtilis* spores expressing surface-exposed r*Pf*CSP induced a significant improvement in anti-r*Pf*CSP IgG levels in mice sera after nasal administration. Additionally, no cross-reactivity between the r*Pf*CSP and the serum from the mice immunized with only *B. subtilis* was observed, which confirms that the observed increase in the lgG level of the anti-r*Pf*CSP in r*Pf*CSP + SBsKO7 group was due to the adjuvant properties of the *B. subtilis* spores. Similar findings have been reported by previous studies^8,9,11,28,29^.

As such, the present study corroborates with others, and demonstrates the ability of the *B. subtilis* spores to induce high levels of serum IgG against vaccine antigens through nasal immunization^9,14,15,30^. Song et al. (2012)^14^ observed higher levels of systemic IgG in mice nasally immunized with *B. subtilis* spores adsorbed with H5N1 virus when compared to animals that received the inactivated virus alone. Futhermore, Lee et al*.* (2010)^28^ also found that mice immunized with *B. subtilis* spores expressing the bovine rotavirus VP6 protein were able to produce increased serum levels of anti-VP6 IgG and anti-VP6 fecal IgA antibodies, while the animals immunized with the VP6 protein alone induced only serum IgG antibodies. In addition, the antibodies produced were able to protect the mice when challenged with rotavirus. High levels of specific antibodies were also found in animals nasally immunized with *B. subtilis* expressing Ig85 and Ag85B antigens from *Mycobacterium tuberculosis*^29^. These studies demonstrated the great potential of the *B. subtilis* spores as a nasally delivered vaccine adjuvant.

Circulating antibodies are considered critical mediators of effective immunity against malaria parasites during sporozoites stage^3^. The follow-up of anti-*Pf*CSP antibodies levels in blood serum is crucial to determine whether the potential adjuvant can promote long-term humoral response. The present study was the first one to follow the antibody response after immunization with a *B. subtilis* spores-based antigen for 250 days, which made it possible to observe and compare the intensity of the murine humoral response. The antibody responses elicited by *B. subtilis* spores were faster, greater and longer (high antibody titers over 250 days) than those observed in the control groups. Increased anti-r*Pf*CSP IgG titers was observed between 14 and 21 weeks post-immunization.

The pattern of humoral responses induced by platform-based vaccines depends on many factors such as the nature and immunogenicity potency of an antigen, immunization delivery strategy and animal model. Mou e*t al.* (2016) orally immunized chickens using *B. subtilis* recombined with the H5N1 protein of the avian influenza virus. The highest serum antibody levels were observed after 3–5 weeks post-immunization^31^. Jelínková et al*.* (2021) reported the presence of serum anti-*Pf*CSP IgG antibodies even after almost two years post-immunization with non-infectious virus particles expressing *Pf*CSP. In this case, the immunization was more effective when they added an adjuvant. This highlights once more the importance of the search for new adjuvant for enhancing the quality of the immune response induced by different vaccine approaches, as described previously^32,33^.

The immunization strategies used in this study induced mainly high serum levels of IgG2b and IgG2a subclasses, followed by IgG1, and low levels of IgG3. The use of *B. subtilis* spores did not interfere in the IgG subclass pattern produced, but it did enhance the levels of antibody titers. The pattern of antibody immune responses continued unaltered until day D250, which suggests that our spore-based approach induced a mixed Th1/Th2 immune response profile, since IgG2a/b are Th2-related isotypes, while IgG1 is associated with the Th1-type response ^34–36^. Santos et al*.* (2020)^13^ reported a mixed Th1/Th2 response generated after using TTFC antigen adsorbed on nasally administered *B. subtilis* spores. De Souza et al*.* (2014)^11^ observed the induction of Th1-dependent IgG2a or Th2-dependent IgG1 antibodies when p24 HIV protein were co-administered with *B. subtilis* spores.

A possible explanation for the modulation of the immune response induced by *B. subtilis* spores is that it promotes an efficient and direct antigen presentation via MHC class I/II and, leading to a balanced humoral and cellular response^17,36,37^. However, in this study, we did not assess cellular immunity induced by the *B. subtilis* spore-based vaccine strategy used.

In humans, production of cytophilic antibodies, IgG3 and IgG1, as observed in the Th1 response is reported to be associated with protection in malaria^38–41^. In mice, high levels of IgG2a/b characterize a Th1 response, since it presents a cytophilic function, acting in complement fixation and pathogen opsonization and, promoting a more efficient fagocytosis than IgG1^34,35^. The results observed in this study demonstrated the improvement in the production of cytophilic antibodies after the use of *B. subtilis* spores as an adjuvant and antigen carrier.

## Conclusion

We present here the first evaluation of the use of a *Bacillus subtilis* spore-based adjuvant/antigen carrier for a malaria vaccine strategy and the humoral response induced by this approach over a period of 250 days of follow-up. The data indicate that the use of this spore could stimulate a greater, faster, and longer antibody response against the *Pf*CSP antigen. In addition, this vaccine approach is very promising because it may induce a balanced Th1/Th2 immune response. Additional studies are necessary in order to reveal the cellular immunity involved in the immune response elicited by the recombinant *B. subtilis* spores expressing *Pf*CSP.

## Supplementary Information

Supplementary Information.
