# Supplementary material for: Bacillus subtilis spores as delivery system for nasal Plasmodium falciparum circumsporozoite surface protein immunization in a murine model
Source: Sci Rep. 2022 Jan 27;12:1531. doi: 10.1038/s41598-022-05344-2 (PMC8795416; doi:10.1038/s41598-022-05344-2)
Supplement: Supplementary file 1 — Supplementary Information. [file 41598_2022_5344_MOESM1_ESM.pdf]

Supplementary data

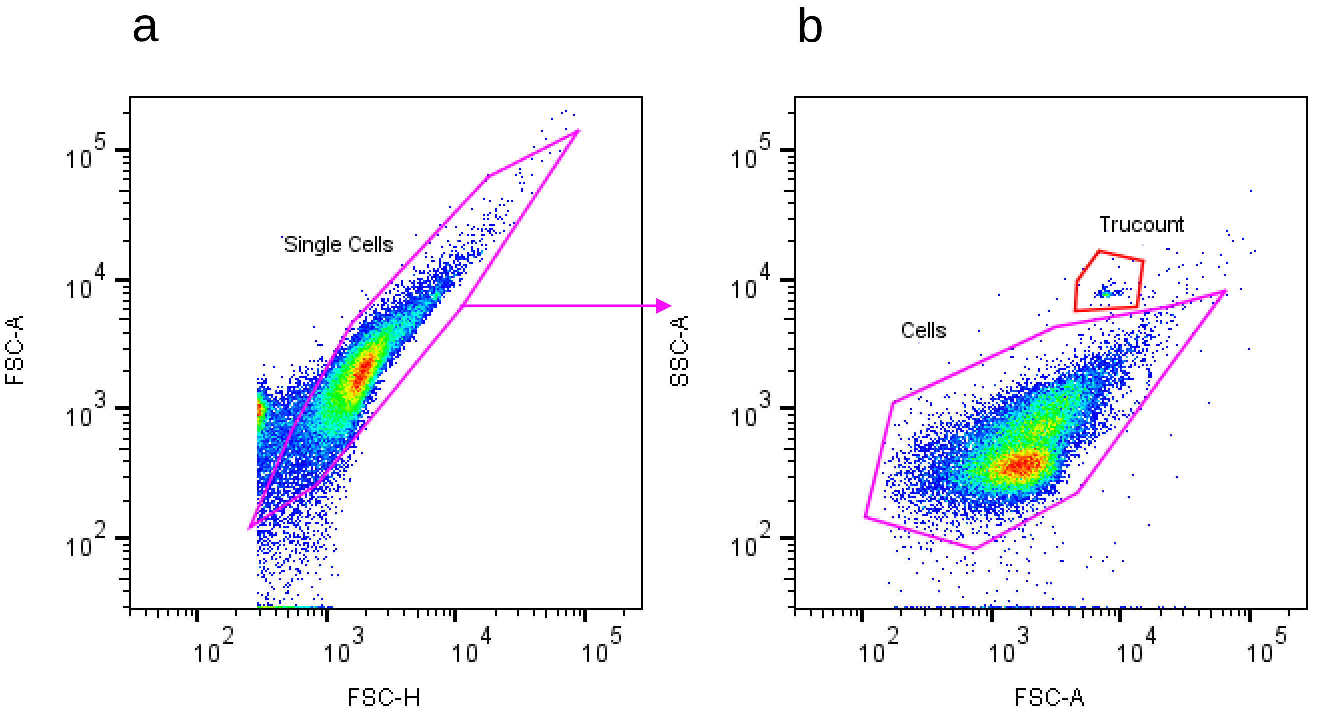

Supplementary Figure 1. Flow cytometry *Bacillus subtilis* spore quantification. (a) Determination of single cells , (b) cells of *Bacillus subtilis* gates

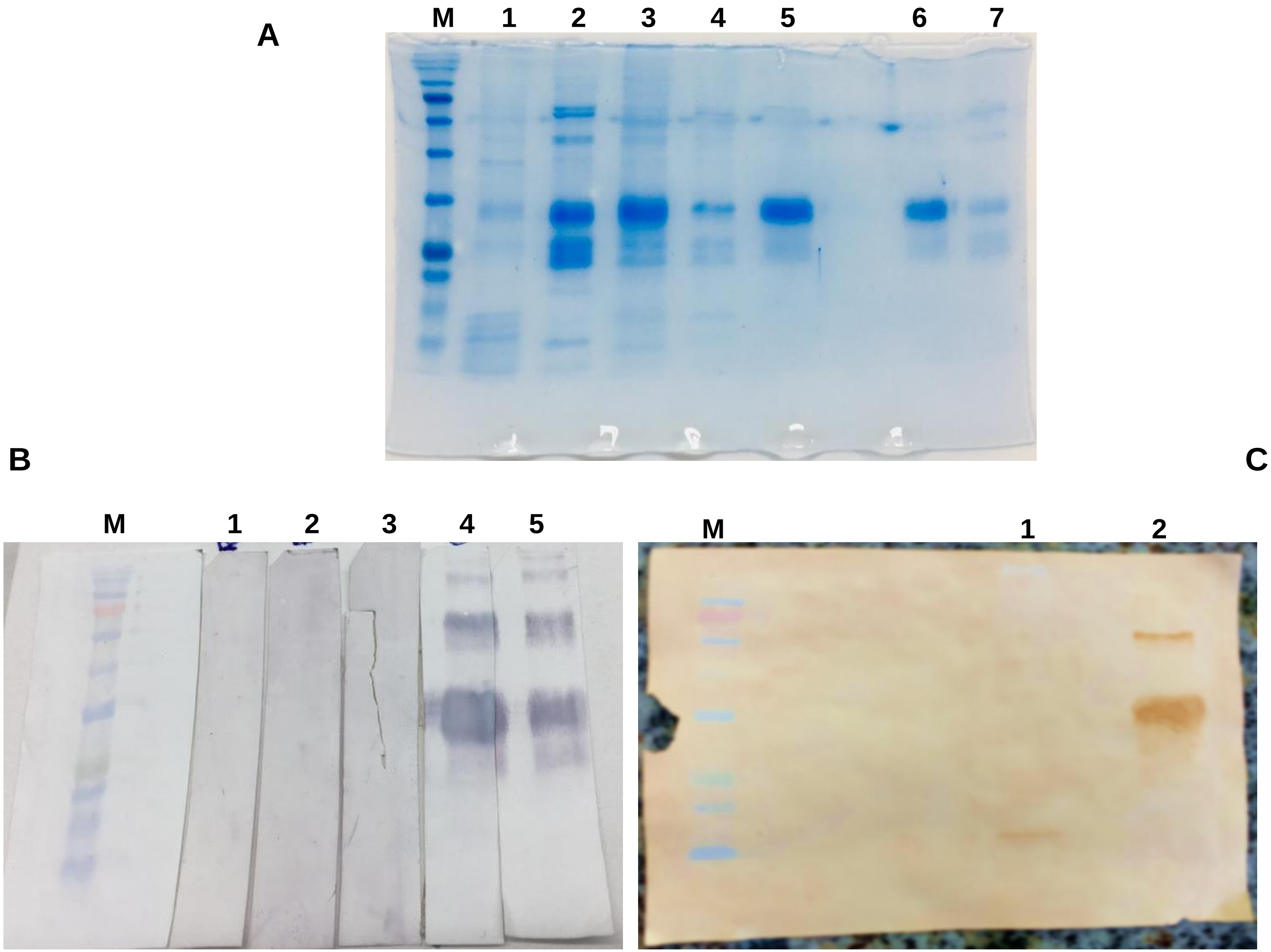

Supplementary figure 2 - Affinity chromatography and western blot analysis of rPfCSP.

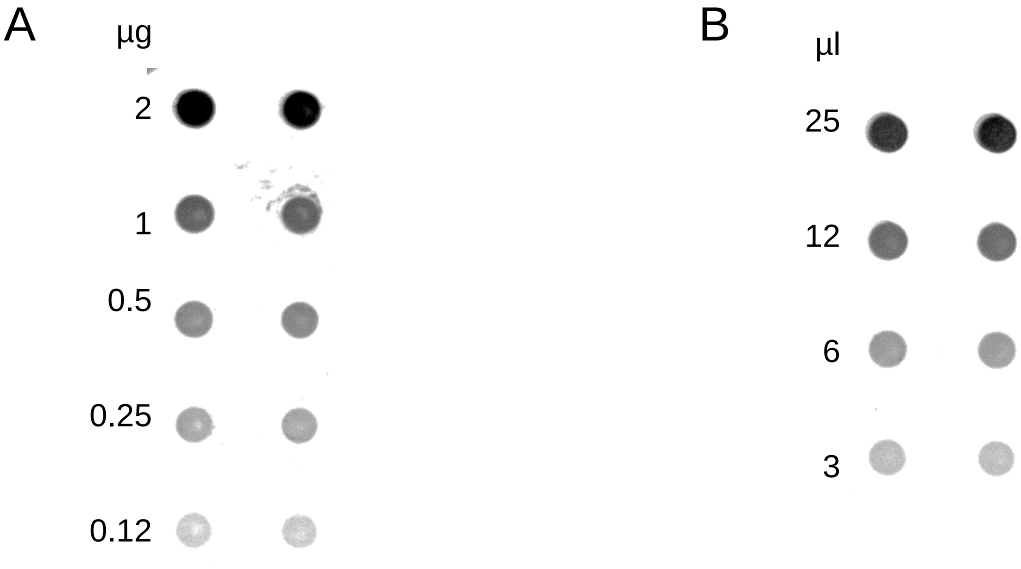

C

#	Label	Vol (Int.)	Local bg. Corr. Vol.	Area	Rf	Density
1	25 µl	91,367,068	34,555,918	2,16	0,056	42,299
2	12 µl	72,266,918	24,694,261	1,932	0,237	37,405
3	6 µl	58,793,936	13,760,604	1,932	0,419	30,431
4	3 µl	35,779,565	4,648,407	1,302	0,611	27,48

#	Local bg. Corr. Den.	% band purity	% lane purity	rolling bg. Corr. Vol.	Rel. quant. (w/RB corr. Vol.)
1	15,998	39,336	0,001	40,440,320	0,86
2	12,781	28,336	0,001	29,131,776	0,626
3	7,122,466	15,913	NA	16,359,680	0,352
4	3,570,206	7,201	NA	7,403,264	0,159

Supplementary Figure 3. Dot blot analysis. A- Standard protein curve; B- Supernatant of coupling method; C- iBright analysis software results.

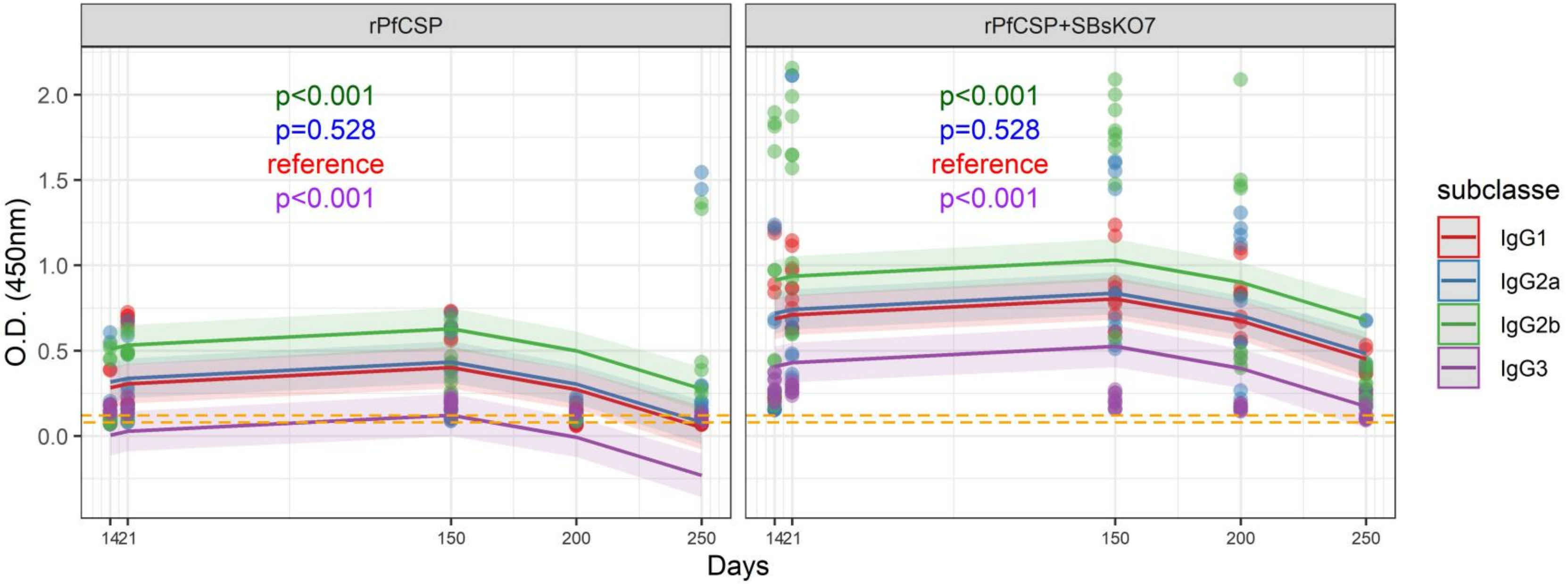

Supplementary Figure 4. Analysis of subclasses by animal groups.

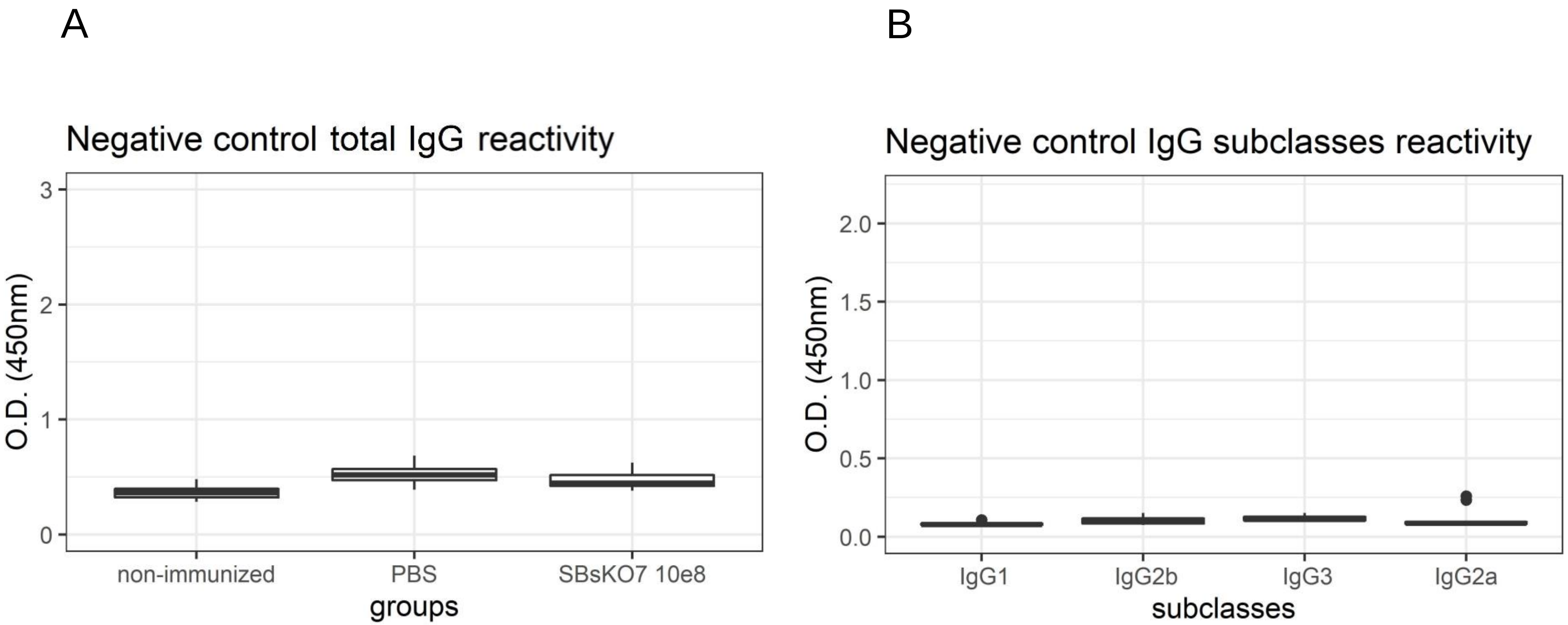

Supplementary Figure 5. Box plot representation of negative control ELISA reactivity. **A:** Reactivity of Negative control total IgG. **B:** Reactivity of negative control IgG subclasses.
